# Current Photoactive Molecules for Targeted Therapy of Triple-Negative Breast Cancer

**DOI:** 10.3390/molecules26247654

**Published:** 2021-12-17

**Authors:** Cristina J. Dias, Luisa Helguero, Maria Amparo F. Faustino

**Affiliations:** 1 LAQV-REQUIMTE, Department of Chemistry, University of Aveiro, 3810-193 Aveiro, Portugal; cristina.jesus.dias@ua.pt; 2 Department of Medical Sciences, Institute of Biomedicine (iBiMED), University of Aveiro, 3810-193 Aveiro, Portugal; luisa.helguero@ua.pt

**Keywords:** triple-negative, breast cancer, TNBC, photodynamic therapy, photothermal therapy, chemotherapy chlorin-based molecules

## Abstract

Cancer is the second leading cause of death worldwide; therefore, there is an urgent need to find safe and effective therapies. Triple-negative breast cancer (TNBC) is diagnosed in ca. 15–20% of BC and is extremely aggressive resulting in reduced survival rate, which is mainly due to the low therapeutic efficacy of available treatments. Photodynamic therapy (PDT) is an interesting therapeutic approach in the treatment of cancer; the photosensitizers with good absorption in the therapeutic window, combined with their specific targeting of cancer cells, have received particular interest. This review aims to revisit the latest developments on chlorin-based photoactive molecules for targeted therapy in TNBC. Photodynamic therapy, alone or combined with other therapies (such as chemotherapy or photothermal therapy), has potential to be a safe and a promising approach against TNBC.

## 1. Breast Cancer

### 1.1. Overview

In 2020, data from the International Agency for Research on Cancer revealed that breast cancer (BC) has the highest incidence (24.5%) and mortality rate (15.5%) in women worldwide [1]. The total number of women affected by BC is projected to increase by 50% by 2040 [2].

BC is a heterogeneous disease, which comprises three major different histological subtypes, defined based on the expression of hormone receptors (oestrogen receptor alpha (ER) and progesterone receptor (PR)), and epidermal growth factor receptor 2 (HER2) [3,4]. Thus, BC is divided into ER- and/or PR-positive, HER2-positive (HER2+) and triple-negative breast cancer (TNBC). Current targeted therapy can be used in ER-/PR-positive tumours, which are susceptible to hormonal therapy, and HER2-positive tumours, which can be treated with anti-HER2 antibodies. On the other hand, TNBC lacks expression of ER, PR and HER2 receptors, and is amongst the most aggressive BC subtypes, with no current satisfactory therapy [3,4,5]. BC subtypes can also be classified according to their gene expression signatures. This molecular classification includes a variety of subtypes with correlation to the histological subtypes described above, being the most common luminal A (ER- and/or PR-positive and HER2-negative), luminal B (ER- and/or PR-positive and HER-positive, as well as high Ki67 expression), HER2 overexpression (ER- and PR-negative and HER2-positive) and a group of tumours that lack ER, PR and HER2 expression—amongst which, the basal-like subtype (expression of basal markers, such as keratins 5, 6, 14 and 17 and epidermal growth factor receptor (EGFR/HER1)) represents 60–90% of TNBC cases [3].

### 1.2. Scope of the Review

This review aims to provide the latest advances and the current molecules explored for targeted therapy of TNBC, since this is a highly aggressive and recurrent disease. In this sense, the main characteristics of TNBC will be explained, followed by a summary of the current therapeutics used in the clinical management of TNBC and a description of emerging strategies to fight the low efficacy of current treatment, focusing on photodynamic therapy (PDT). PDT is of particular interest, since it has been used with success in the treatment of several types of cancer. The anti-cancer mechanisms of PDT have been extensively reviewed; therefore, this review will briefly summarize the inherent anti-cancer mechanisms of the photodynamic process. Thus, the focus of this work is to describe the chlorin-based molecules, known by their good photophysical and photochemical features, which make these molecules promising for application as photosensitisers (PS) in cancer therapy. Additionally, their photoactivity towards TNBC and the cell death mechanisms will be discussed.

### 1.3. Triple-Negative Breast Cancer

TNBC is diagnosed in ca. 15–20% of BC cases and this subtype is more prevalent in pre-menopausal African and Hispanic women [6,7]. Besides ethnicity, other risk factors for TNBC include age—since younger women are more predisposed to develop TNBC—being a carrier of inactivating BRCA1 mutations and obesity, among others [3,8]. TNBC is a highly heterogenous and very aggressive cancer, characterized by high grade and large sized lymphocyte infiltration and reduced response to chemotherapy. Moreover, due to the absence of hormonal receptors, targeted systemic therapies (trastuzumab and hormonal therapies) are ineffective [9]. During diagnosis, the manifestation of early visceral metastasis and lymph node involvement is frequent; this results in a reduced survival rate and higher incidence of relapses due to metastasis appearing mainly in lungs and brain [6]. Patients diagnosed with TNBC have a 5-year survival rate of 62% [10], whereas patients with advanced TNBC have an average survival time of 1 year [6]. Even with the current mammography screening programs, the use of magnetic resonance imaging (MRI) and ultrasound, TNBC detection at early stages is difficult, because, due to their aggressiveness, they may develop very quickly and be found in advanced stages, when the tumour lesions have a size larger than 2.5 cm [6,11,12]. Thus, it is highly important to find innovative alternative therapies for the treatment of TNBC that bring a better prognosis for patients.

### 1.4. Triple-Negative Breast Cancer Therapeutics

The standard treatments currently used in TNBC are chemotherapeutic drugs used in combination with surgery and/or radiation therapy [13]. Chemotherapeutic drugs can be divided into anthracyclines, taxanes and platinum compounds, and the administration of any of these drugs depends on the patient’s clinical presentation, including the tumour stage [6,14]. In some cases, the efficiency of chemotherapy on TNBC is low or null because the tumours exhibit resistance to the drug. The resistance mechanisms observed in TNBC are related with the following: overexpression and/or activity of ATP-binding cassette (ABC) transporters; overexpression of β-tubulin II subunit; mutations in DNA repair enzymes; alterations in genes involved in apoptosis; drug inactivation/detoxification; nuclear factor (NF)-κB signalling pathway; KIF14-mediated AKT phosphorylation [15]. Therefore, while chemotherapy may be effective for reducing a large amount of the tumour volume, tumour cell subpopulations that remain resistant to chemotherapy and radiotherapy may originate metastasis and relapse. The ineffectiveness of chemotherapy and radiation, the high risk of relapse in TNBC patients and the serious side effects of these therapies have prompted the development of more advanced, safe and effective therapeutic strategies. Immunotherapies, for instance, are used in clinical treatment of TNBC [16,17], since this type of breast cancer is very immunogenic [18,19]; additionally, EGFR/HER1, receptors overexpressed in TNBC cells, are being explored for the development of targeted therapies, in fact, phase II clinical trials with EGFR-targeted therapy have shown prolonged progression-free survival and overall survival in metastatic TNBC patients [20]. Moreover, another receptor was explored in preclinical studies—insulin growth factor-1 receptor (IGF-1R)—which also high expressed in TNBC cells [21]. IGF-1R-targeted therapy reveals tumour growth inhibition in TNBC tumour graft MC1 [21]. Besides, poly(adenosine diphosphate (ADP)- ribose) polymerase (PARP) and mammalian target of rapamycin (mTOR) inhibitors have been explored as potential therapeutic targets for TNBC [15]. Photothermal therapy (PTT) and photodynamic therapy (PDT) are other treatment modalities that have attracted considerable attention for TNBC treatment and are being explored in preclinical studies [22,23], as can be seen in later sections.

## 2. Photothermal Therapy

PTT is an emerging therapeutic strategy and consists of the use of photothermal agents that are stimulated by a near-infrared laser (which can penetrate a couple of centimetres in live tissues) to generate heat triggering hyperthermia (41–47 °C), leading to thermal ablation of tumours [22,24,25,26]. This therapy has the ability to eliminate cancer cells in primary tumours or local metastasis in early stages; moreover, in preclinical models, PTT has the ability to reduce distant metastasis when combined with other therapies, such as chemotherapy [26,27,28]. The photothermal agents usually used in PTT are inorganic nanomaterials, such as metal nanoparticles [29,30,31], graphene oxide [32] and carbon nanotubes [33]. PTT outstands with its high specificity, minimal invasiveness and precise spatial–temporal selectivity [26,28,34,35]. Additionally, since different mechanisms of action are related to PTT and PDT action, such therapies could be used alone or combined, to obtain a synergistic effect against tumour cells and consequently obtain improved effectiveness in eliminating cancer cells [6].

## 3. Photodynamic Therapy

**Figure 1:**
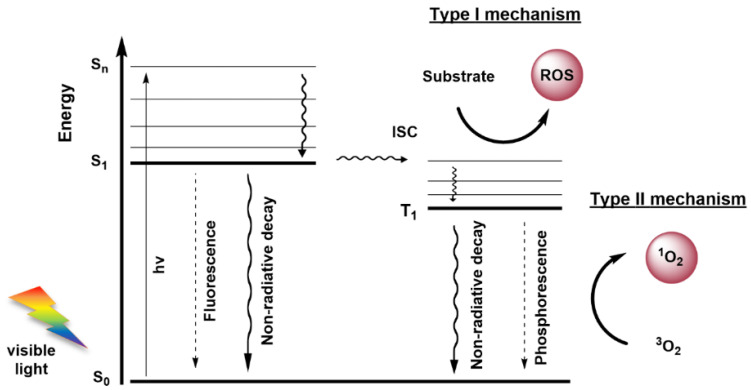
Representation of modified Jablonski diagram.

Photodynamic Therapy (PDT) stands out as a non-invasive modality to treat neoplasia and inactivates of a wide range of microorganisms such as bacteria, viruses and fungi, among others [36,37,38]. PDT has been used in the treatment of several types of cancer, including superficial tumours (e.g., basal cell carcinomas (BCC), head and neck tumours) and tumours accessible by endoscopy (e.g., lung and oesophageal cancer). The photodynamic process requires a photoactive molecule (known as a photosensitizer (PS)), visible light and dioxygen. As soon as the PS is exposed to light in the presence of dioxygen, the formation of ROS is triggered [36,37,38,39,40]. These include singlet oxygen (^1^O_2_), hydrogen peroxide (H_2_O_2_) and free radicals (e.g., OH·, O_2_^−^·), which are highly reactive oxidative species that react with biomolecules to cause cell death. Other excitation sources have been explored as an alternative to the conventional visible light, such as Cerenkov radiation (electromagnetic radiation produced when a charged particle passes through a dielectric medium at a greater velocity than the phase velocity of light in that medium, generating local polarization) [41,42], chemiluminescence (transfer of energy from the chemiluminescent emitter to the PS) [43,44] and X-rays (an energy transducer is used to transfer X-rays to optical luminescence and start radiotherapy and PDT) [45,46]. The process of ROS formation in PDT is mediated by two mechanisms—Type I and Type II mechanisms (Figure 1) [36,37,38,39]. In the photodynamic process, irradiation of PS with light causes the PS promotion from its ground state (S_0_) to an excited singlet state (S_n_). However, the PS can decay to its lower energetic level (S_1_) by internal conversion, and in this singlet excited state (S_1_), the PS can release the excess of energy by fluorescence emission or by a non-radiative decay, returning to S_0_. Alternatively, the PS can be converted in an excited triplet state (T_1_) through an intersystem crossing (ISC) process. In T_1_ state, the PS can decay to S_0_ by phosphorescence emission (forbidden transition), or by the aforementioned mechanisms: Type I—when the PS in T_1_ state can donate or accept electrons from other substrates to form radicals that react with dioxygen; or Type II—in which the PS (T_1_) transfers energy directly to dioxygen in the triplet ground state (^3^O_2_), converting it in highly oxidant ^1^O_2_ [36,37,38,39].

### 3.1. Photosensitizers

**Figure 2:**
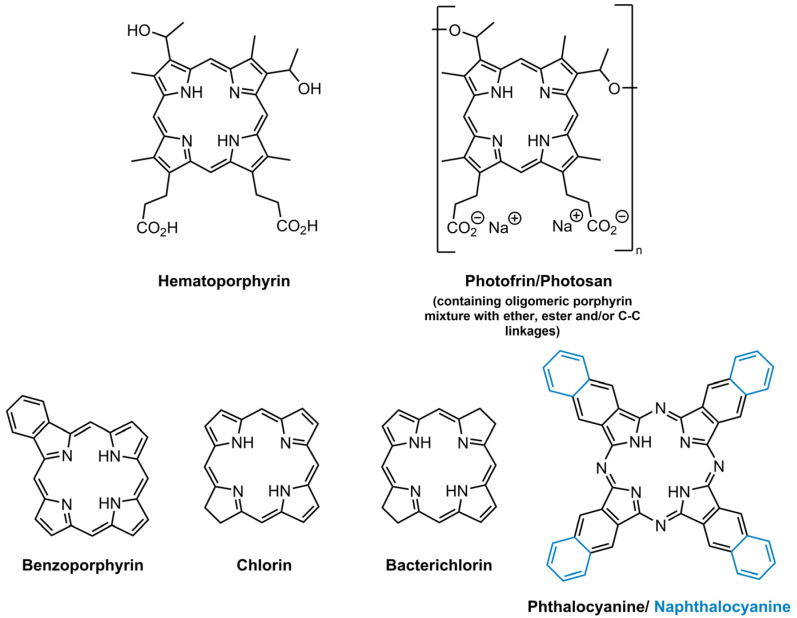
Structures of some first generation PS and the core structure of second generation PS.

The ideal structure of a PS depends largely on its application, but some characteristics are known to bring a positive impact to their efficiency. Namely, the following: stability; selectivity towards neoplasic tissues and rapid clearance from healthy tissues; amphiphilicity; minimal cytotoxicity in the dark; high singlet oxygen quantum yield; high absorption on the therapeutic window (600–800 nm—where the light can penetrate efficiently in the tissues); easy availability and synthesis; high chemical purity [37,39,47]. The PS used in clinical applications of antitumoural PDT, which are already approved, are in general hematoporphyrin derivatives, considered as the first generation PS (Figure 2). Photofrin^®^ and Photosan^®^ formulations (a porphyrin mixture with monomers, dimers and oligomers entities) are some of first generation PS that are used presently in clinical PDT. Nevertheless, patients treated with these market PS exhibited high cutaneous photosensitivity and they have low absorption in the therapeutic window, limiting their application to small tumours [37,39,47].

This prompted the scientific community to develop PS with better photodynamic efficiency, which are characterized by single chemical entities, high chemical purity, good absorption in the wavelength range between 600–800 nm, higher singlet oxygen quantum yield and minimal side-effects [37,39,47]. These PS are labelled as second generation PS and belong to different groups, such as benzoporphyrins, chlorins, bacteriochlorins, phthalocyanines and naphthalocyanines (Figure 2). Although this second generation PS, in general, exhibited better selectivity to tumour tissues, their poor solubility in physiologic medium is a limiting factor. Aiming for the development of a PS with a higher affinity to, or targeting of, the tumour tissues, third generation PS were developed, which are based on second generation PS that are combined with other biomolecules, such as monoclonal antibodies, liposomes and carbohydrates, among others [39,47]. These biomolecules allow the improvement of photodynamic efficiency by enhancing the selectivity towards neoplastic cells and improving solubility.

### 3.2. Anti-Cancer Mechanisms of Photodynamic Therapy

PDT consists of topical or systemic administration of the PS and its selective accumulation in the tumoural tissue, followed by irradiation with an appropriate light wavelength [48]. After light activation in the presence of dioxygen, the formation of ^1^O_2_ takes place. This highly reactive specie is responsible for the tissues damage. Since the lifetime of ^1^O_2_ is very short, damage occurs close to the PS location where ^1^O_2_ was generated [48]. The PS usually localizes in cytoplasmic, mitochondrial and lysosomal membranes and in Golgi apparatus and endoplasmic reticulum; due to its hydrophilic nature, these PS are internalized by endocytosis into the cells [49]. Depending on the severity of the photodamage, it may be permanent or repaired, and different stress responses are triggered after PDT to repair the damage [50]. The integrated stress response consists of the unfolded protein response (UPR) and the antioxidant response. The UPR consists of three branches (inositol-requiring protein 1α/X-box-binding protein 1α (IRE1α/XBP1), protein kinase RNA-like endoplasmic reticulum kinase/eukaryotic translation initiation factor 2-α (PERK/eIF2-α) and activating transcription factor 6 (ATF6)). These branches become active when the cell senses endoplasmic reticulum stress and protein misfolding. This leads to transient inhibition of protein translation (through PERK/eIF2-α) and activation of transcription factors (XBP1s and ATF6) that activate pathways aiming to re-establish cell homeostasis [51]. The antioxidant stress response is activated by oxidative stress and results in activation of the erythroid 2-related factor (Nrf2) transcription factor, which, in turn, stimulates the expression of enzymes capable of eradication of oxidized biomolecules and electrophilic agents [50,52]. When the stress response fails, or the oxidative stress is more intense and prolonged, the mechanisms of cell death are activated [50]. Apoptosis, necrosis and autophagy are the most explored cell death pathways in PDT [48,49,50]. Depending on the localization and/or the concentration of PS vs. light dose, PDT can induce different forms of cell death simultaneously in the same tissue. Although apoptosis is the most common cell death mechanism seen in PDT [48,49,50,53], it can be divided in two main pathways, the intrinsic or mitochondrial pathway and the extrinsic or death receptor pathway [49,50]. The intrinsic pathway is associated with intracellular and extracellular disturbances concerning the permeabilization of the outer membrane of mitochondria, and is the main pathway in the context of PDT. On the other hand, the extrinsic pathway is related to the disturbances at the extracellular environment that are detected by plasma membranes receptors [49,50]. These pathways have the activation of caspase cascades in common, which stimulates several biochemical processes that result in apoptosis [49]. Necrosis, or accidental cell death, on the other hand, can occur with high doses of light and/or PS, and with loss of membrane integrity and cell lysis with PDT [49,50,54]. This mechanism is also related with PS, having a tropism for the cell membrane; in fact, some studies demonstrate that short incubation periods allow PS to accumulate preferentially in the plasma membrane, so that after PDT results in cell death by necrosis, instead, long incubation periods will result in apoptosis mechanism [49,50]. These differences in the stimulated death mechanisms are important, as necrosis induces an inflammatory response, while this does not occur with apoptosis [55]. Autophagy consists of the degradation and recycling of damaged macro-proteins or organelles that are retained into lysosomes [49,53]. PDT-induced ROS causes the formation of autophagosomes, responsible for transporting cellular components for degradation to the lysosomes [56]. Autophagy has a cytoprotective role but can be lethal, depending on the nature of the PS, the PDT dose light, the PS concentration and the cells [49,57]. Since autophagy often leads to cell death through apoptosis or necrosis, it has a less significant role in PDT, except if cytoprotective autophagy is activated [50]. Recently, paraptosis was reported, which consists of a new cell death type associated with the cells’ response to PDT [58,59,60]. This process seems to occur when the PS causes photodamage in the endoplasmic reticulum and seems to be a consequence of misfolded endoplasmic reticulum proteins. Additionally, the paraptosis mechanism is characterized by cytoplasmic vacuolization [58,59,60]. However, it is also reported that paraptosis may also be induced by a PS, which causes nuclear photodamage; it was observed that cells which accumulate at the G2/M interface may experience paraptosis, in turn, cells that stay blocked from undergoing mitosis die by apoptosis [60,61]. Nevertheless, the paraptosis mechanism is not yet fully understood, since there are only a few studies reporting paraptosis after PDT [58,60].

Other indirect mechanisms that influence the effect of PDT on tumour vasculature, the immune system and inflammatory responses are described in the literature [57]. Regarding tumour vasculature, the PDT effect depends on the PS pharmacokinetic, and induction of vascular endothelial growth factor (VEGF), cyclooxygenase 2 (COX2) and phosphoinositide 3-kinase (PI3K) molecular pathways were observed in the tumour microenvironment. On the other hand, the efficiency of PDT is associated with the immune response, in the way that PDT causes the destruction of tumour cells with activation of acute inflammatory reaction [39,47,48,56]. The effect of PDT on the immune system is related with signals generation (damage-associated molecular patterns (DAMPs)) after oxidative stress in endoplasmic reticulum, induced by PDT [62,63]. These DAMPS are released after immunogenic cell death is triggered by PDT and are identified by pattern recognition receptors expressed on immune cells, leading to activation of adaptative immunity [50,56,63]. Finally, the inflammatory response is stimulated by lipid peroxidation after PDT, with the release of arachidonic acid metabolites or by vasoactive substances (e.g., components of the complement and clotting cascades, proteinases, peroxidases, ROS, leukocyte chemoattractants, cytokines and transforming growth factor (TGF)-β) [56,57]. These elements promote the stimulation of innate immune cells into the tumour. The inflammatory response is responsible for clearing the debris and recovering homeostasis [56,57].

## 4. Targeted Therapy in Triple-Negative Breast Cancer

Several classes of molecules, such as tetrapyrrolic macrocycles and analogues, including porphyrins [64,65,66,67,68,69,70,71], phthalocyanines [72,73], chlorins [22,23,74,75,76,77,78,79,80,81,82,83,84,85,86], the prodrug 5-aminolevulinic acid (5-ALA) [87,88,89] and other dyes, such as adequately conjugated indocyanine green [90,91,92,93,94], have been explored as PS in TNBC targeted therapy. Chlorins and their analogues are very interesting molecules; besides their natural source and/or semi-synthetic preparation, these molecules exhibit good absorption in the therapeutic window (600–800 nm), which is an important feature for a good candidate for clinical PDT, due to the better light penetration in tissues (≈1–2 cm), which overcomes the light penetration limitation when other photosensitizers are used, such as porphyrins [48,49,95]. It is important to mention that this review will not cover all the papers concerning the chlorin-based molecular formulations, but will describe the different strategies currently used and the phototherapeutic efficiency of these molecules for targeted therapy of TNBC.

**Figure 3:**
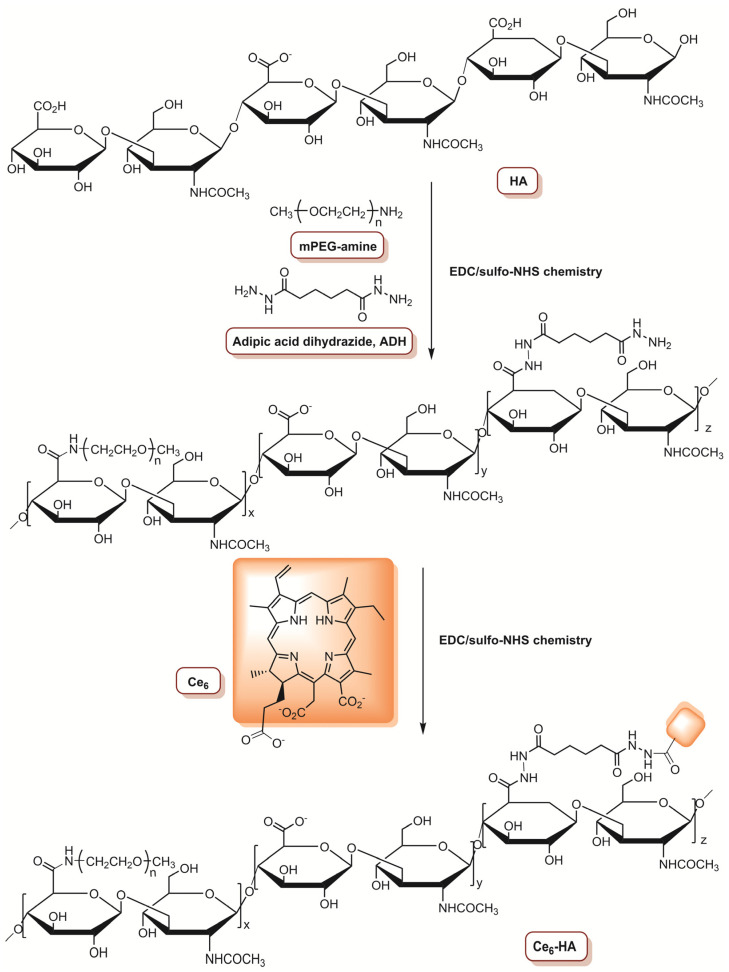
Schematic representation of the preparation of Ce_6_-HA (adapted from Reference [74]).

In 2015, Choi and co-workers [74] reported the development of antitumour drug-loaded polymeric nanoparticles (NP), such as an enzyme-activatable theranostic NP (EAT@NP), for selective near-infrared fluorescence imaging and dual PDT/chemotherapy. The preparation of EAT@NPs involves the covalent conjugation of chlorin e_6_ (Ce_6_) to a monomethoxypoly(ethyleneglycol)-grafted hyaluronic acid (HA) backbone (Figure 3), followed by self-assembled NPs formation (Ce_6_@HA@NPs). After that, a topoisomerase 1 inhibitor (anticancer drug with low solubility), named camptothecin (CPT), was encapsulated inside Ce_6_@HA@NPs using a dialysis method, resulting in EAT@NPs. The fluorescence of CPT and Ce_6_ was quenched inside EAT@NPs. In this sense, the ability of intracellular hyaluronidase (HAdase) (overexpressed in breast metastatic tumours and responsible for the breakdown of HA backbone) to stimulate the recovery of NIR fluorescence and ^1^O_2_ generation was investigated, as well as the release of CPT. After treatment of EAT@NPs with HAdase, both NIR fluorescence and ^1^O_2_ generation were activated and the degradation of NPs triggered the CPT release. The therapeutic effect of EAT@NPs was evaluated in MDA-MB-231 cells at different concentrations (0–20 μM) of CPT (corresponding to 0–10 μM Ce_6_ equivalent for EAT@NPs) and the cells were irradiated with a CW laser at 670 nm with a total light dose of 10 J cm^−2^ (0.068 W cm^−2^). At a concentration of 1.0 μM of CPT equivalent, without irradiation, free CPT causes a reduction of 12% in the cancer cells viability, while in cells incubated with EAT@NPs, the reduction was 28%. After light exposure, a reduction of 67% in cell viability was observed at the same concentration for cells treated with EAT@NPs. Moreover, when the concentration was increased to 20 μM CPT equivalent, the PDT efficiency was improved, since the cell viability was only 2% for MDA-MB-231 cells treated with EAT@NPs, while without irradiation, the cell viability was 50% and 68% for EAT@NPs and free CPT, respectively. This study confirmed the potential photodynamic/chemo dual therapy of the prepared EAT@NPs. Moreover, confocal microscopy demonstrates that EAT@NPs efficiently enter the cells, which was confirmed by the observation of strong fluorescence signals [74].

**Figure 4:**
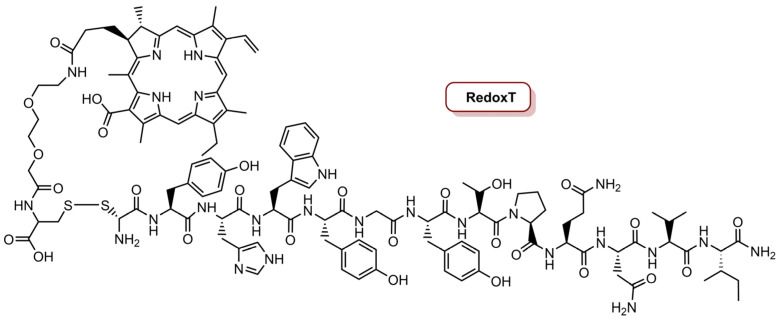
Representation of the structure of redox-responsive specific theranostic (RedoxT) for specific fluorescence imaging and PDT.

As mentioned before, EGFR are overexpressed in the majority of TNBC cells and constitute an important therapeutic target [75,96]. In this sense, in 2016, the same group [75] reported the development of a redox-responsive specific theranostic (RedoxT) for specific fluorescence imaging and PDT of a TNBC cell line, with overexpression of EGFR (Figure 4). The preparation of RedoxT involved the conjugation of chlorin e_4_ (Ce_4_) with the highly specific EGFR-targeting peptide GE11 through disulphide linkers. The target specificity of RedoxT to EGFR was studied in two cells lines with moderate and high EGFR expression, MDA-MB-231 and MDA-MB-468, respectively, and a control cell line PCA-SMCS (primary coronary artery smooth muscle cells) with low EGFR expression was also used. In a concentration of 2.0 µM, RedoxT showed very low intracellular uptake in normal cells, but the uptake was increased with the increase level of EGFR expression. Additionally, the RedoxT preferentially accumulated in the intracellular lysosomes, which allow the cleavage of the disulphide linkers by glutathione. The photoactivity of RedoxT (670 nm CW, 20 J cm^−2^, 0.05 W cm^−2^) revealed an IC_50_ of 0.66 µM in MDA-MB-468 cells and 1.80 µM in MDA-MB-231 cells. On the other hand, when cells were treated with free Ce_4_ and then irradiated, the IC_50_ was 2.22 µM and 2.98 µM for MDA-MB-468 and MDA-MB-231 cells, respectively. This observation confirms that RedoxT activated the PDT effect inside cells after specific internalization into cancer cells. In vivo studies in a xenograft mouse model with MDA-MB-468 cells revealed that RedoxT preferentially accumulates in the tumours rather than in the nearby normal tissues [75].

**Figure 5:**
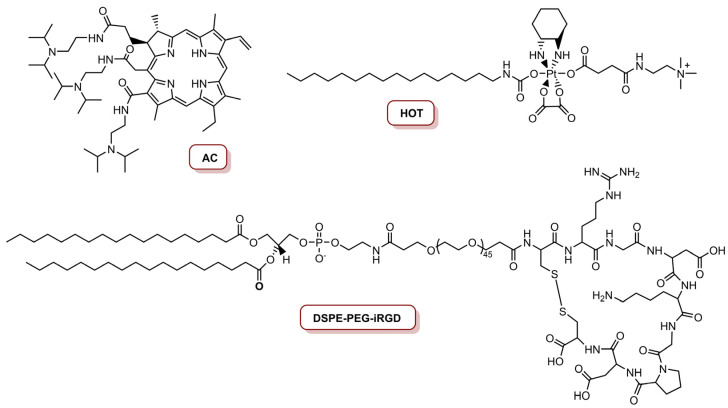
Structures of the acid derivative of Ce_6_ (AC), HOT prodrug and iRGD-conjugated phospholipid (DSPE-PEG-iRGD).

The combination of chemotherapy and PDT led Li and co-workers to develop a multifunctional oxaliplatin (Oxa) prodrug NP for precise imaging and organelle-specific PDT and chemotherapy [76]. The preparation of this NP involved the synthesis of an acid-activatable PS (a derivative of Ce_6_) called AC (Figure 5), through the coupling of three diisopropylethylene diamine (DPA) molecules on each carboxylic group of Ce_6_. Then, Oxa (anti-cancer drug) was oxidized with hydrogen peroxide, and covalently conjugated with hexadecylisocyanate and trimethyleneamine on two heads of the axis, respectively, in order to obtain the stearylamine (SA)-mimicking Oxa prodrug of hexadecyl-oxaliplatin-trimethyleneamine (HOT) (Figure 5). Considering the targeting ability of NPs, iRGD, a target ligand for tumour homing and penetration, was conjugated with 1,2-distearoyl-*sn*-glycero-3-phosphoethanolamine-*N*-methoxy-poly(ethyleneglycol) (DSPE-PEG), resulting in an iRGD-conjugated phospholipid (DSPE-PEG-iRGD) (Figure 5). Finally, the resulting iNP@AC were obtained by self-assembling HOT prodrug, AC and DSPE-PEG-iRGD through a hydrophobic interaction using a film hydration method. Additionally, NPs without iRGD were prepared and called NP@AC, resulting from the self-assemble of HOT prodrug, AC and DSPE-PEG. The photoactivity of the NPs in the mouse cell line 4T1, after laser irradiation at 655 nm for 30 s at an irradiance of 0.25 W cm^−2^ (7.5 J cm^−2^) and 0.63 W cm^−2^ (18.9 J cm^−2^), and at a concentration of 300 nM platinum and 8.0 nM AC, revealed a gradual decreased in cell viability in an irradiance-dependent manner, when cells were treated with AC, iNP@AC and NP@AC, due to the phototoxicity induced by AC. Moreover, the combination of AC-induced PDT and HOT-mediated chemotherapy caused a cell viability reduction of 81% at an irradiance of 0.63 W cm^−2^. The HOT-induced mitochondrial depolarization and DNA damage were together responsible for cell apoptosis. In vivo studies in the lung metastatic 4T1 model revealed that treatment with iNP@AC and laser irradiation exhibited the slowest tumour growth rate with the tumour, which was due to necrosis and apoptosis of the tumour cells, triggered by ROS generation, induced by AC and cytotoxicity stimulated by HOT, respectively [76].

**Figure 6:**
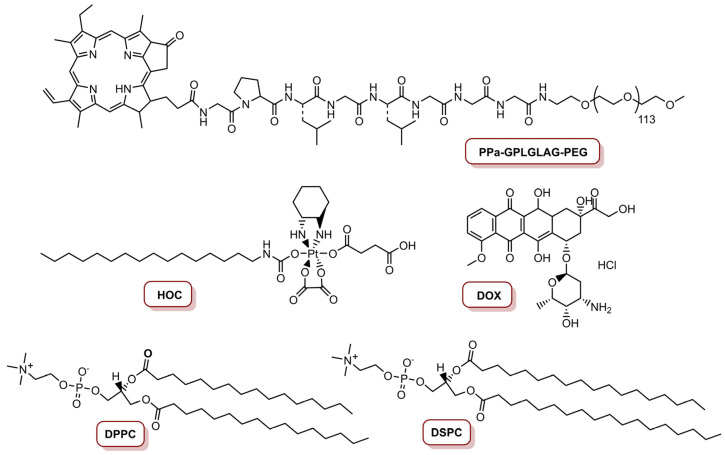
Structures of PPa-GPLGLAG-PEG, HOC prodrug, DOX and the DPPC and DSPC phospholipids used to prepare liposomal vesicles.

The same group, in 2017, reported a programmed multi-responsive liposomal vesicle for enhanced tumour penetration [77]. The preparation of these vesicles involved, firstly, the conjugation of pyropheophorbide *a* (PPa) at the end of polyethyleneglycol (PEG) chain through a GPLGLAC heptapeptide spacer (matrix metalloproteinases (MMP)-2/9-liable linker), resulting the PPa-GPLGLAG-PEG. Then, the enzyme, the light and temperature multi-sensitive liposomal (ELTSL) vesicles were prepared by film hydration and stepwise extrusion in a 1,2-dipalmitoyl-*sn*-glycero-3-phosphocholine (DPPC):1,2-distearoyl-*sn*-glycero-3-phosphocholine (DSPC):PPa-GPLGLAG-PEG molar ratio of 72:24:4 (Figure 6). An MMP-2 insensitive LTSL vesicle was also prepared and used as control. The ELTSL is expected to accumulate selectively in the tumour through the enhanced permeability and retention effect, and then subsequently strip the PEG corona through MMP-2-mediated cleavage of peptide spacer for deeper penetration inside the tumour. The vesicles were loaded with two first-line chemotherapeutics, one was a lipophilic oxaliplatin prodrug of hexadecyl-oxiplatin carboxylic acid (HOC) and the other was doxorubicin (DOX), in the lipid membrane and in the core of the ELTSL, respectively (Figure 6). Biological evaluation in different cell lines (4T1, MDA-MB-231 and MDA-MB-468) showed that ELTSL-HOC/DOX and LTSL-HOC/DOX (both prepared at a HOC to DOX load molar ratio of 5:1 and a PPa concentration of 1.0 µM) exhibited higher cytotoxicity than free DOX, HOC and single drug-vesicles. After irradiation with a laser at 670 nm, an increase in photocytotoxicity for ELTSL-HOC/DOX was observed, possibly due to the previously cumulative effect of DOX and HOC and the combination of therapeutic effect between chemotherapy and PDT. The cellular uptake and tumour penetration was shown to be higher for ELTSL than for LTSL. In vivo studies in an orthotopic 4T1 tumour model, with laser irradiation for 10 min at an irradiance of 0.4 W cm^−2^ (total light dose of 240 J cm^−2^), revealed that ELTSL-HOC/DOX efficiently supressed the growth of the tumour because of the synergistic effect of chemotherapy and PDT, mediated by HOC/DOX and ELTSL, respectively [77].

**Figure 7:**
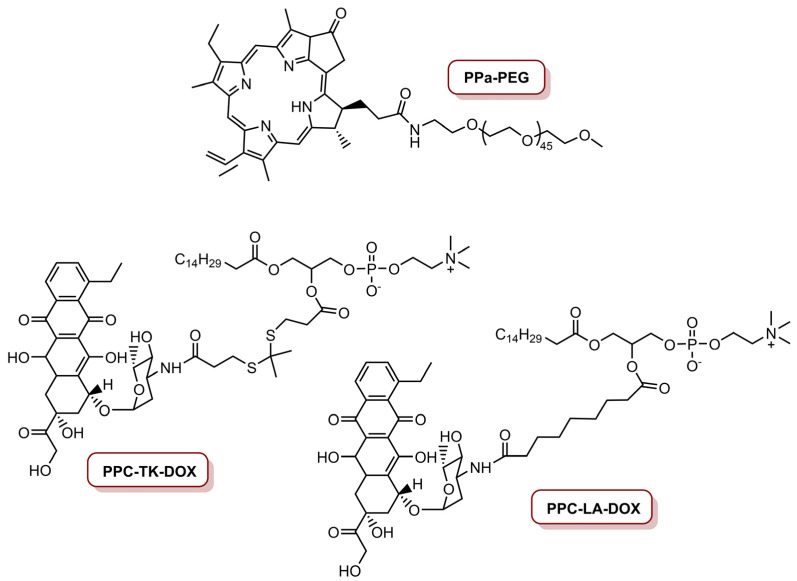
Molecules involved in the preparation of ROS-activatable doxorubicin theranostic prodrug vesicles, PPa-PEG, PPC-TK-DOX and PPC-LA-DOX, respectively, for RADV and RIADV.

This group also developed a ROS-activatable DOX theranostic prodrug vesicle (RADV) for local–regional therapy of metastatic cancer [78]. This nanoplatform was prepared starting with the conjugation of PPa on the amino group of methoxy-PEG-NH_2_ via amide bond, resulting in PPa-PEG (Figure 7). Then, DOX was linked on the hydroxyl group of 1-palmitoyl-2-hydroxy-*sn*-glycero-3-phosphocoline (PPC) with a ROS-cleavable thioketal (TK) spacer to obtain the ROS-activatable DOX prodrug (PPC-TK-Dox) (Figure 7). Finally, this phospholipid-mimicking PPC-TK-DOX was self-assembled with both PPa-PEG—an unsaturated phospholipid 1,2-dioleoyl-*sn*-glycero-3-phosphocholine (DOPC)—and cholesterol, using film hydration and membrane extrusion method to prepare the RADV nanoplatforms. The incorporation of PPa allows the nanoplatform to generate fluorescence and photoacoustic signals to guide NIR laser irradiation. This leads to generation of ROS that consequently trigger DOX at the tumour site for local–regional combined chemotherapy and PDT. Additionally, an ROS-inactivated DOX prodrug vesicle (RIADV) was prepared with PPC-LA-DOX, which corresponds to the conjugation of DOX on PPC with a lepargylic acid (LA) spacer (Figure 7). Both RADV and RIADV demonstrate similar ROS generation; thus, the chemical structure did not seem to affect the photochemical property of the nanoplatforms. The biological efficiency of these nanoplatforms in 4T1 cells demonstrate that both nanoplatforms were not cytotoxic. Under laser irradiation at 670 nm, at an irradiance of 0.1 W cm^−2^ during 2 min (total light dose of 12 J cm^−2^) and in a DOX concentration of 1.0 µM, cell viability decreased to 12.5% for RADV, and remained in 62.5% for RIADV. Laser irradiation of cancer cells treated with RADV induced 75.8% apoptosis or necrosis. In vivo studies revealed that RADV accumulates in the tumour and induces ROS generation under laser irradiation. Furthermore, RADV (DOX dosage of 5.0 mg Kg^−1^) was able to inhibit 85% of tumour growth with localized laser irradiation at an irradiance of 0.2 W cm^−2^ (total light dose of 60 J cm^−2^). On the other hand, RIADV only presents an inhibition of ca. 60% of tumour growth, as a result of PPC-LA-DOX prodrug was insensitive to ROS. RADV was also highly efficient to inhibit lung metastasis of 4T1 cells. Thus, the combined therapeutic effect of PDT and chemotherapy highly improved the efficiency of RADV to inhibit both tumour growth and lung metastasis [78].

Combined PTT and PDT has been investigated due to its numerous advantageous aspects, such as its high spatial selectivity, its non-invasive nature and its minor drug resistance [22,97]. Oh and co-workers [79] developed NPs, based on Ce_6_ conjugated with copper sulfide (CuS) for combined PTT/PDT. Polyethylenimine-coated CuS NPs (PEICuS) were grafted to Ce_6_ through an amide linkage to the amine groups of CuS NPs, to allow the formation of Ce_6_@PEICuS NPs. Biological evaluation of these Ce_6_@PEICuS NPs in MDA-MB-231 cells revealed that at a concentration of 200 µg mL^−1^, on independent treatments of PDT (670 nm, 100 mW cm^−2^, 60 J cm^−2^) and PTT (808 nm, 2.0 W cm^−2^, 1200 J cm^−2^) achieved, respectively, reductions of 55% and 41% in cell viability. However, when PDT and PTT were combined in a single treatment (under the same conditions) the efficiency increased significantly, with a cell viability decrease of 84%, confirming the ability to produce ROS when PDT was performed and thermal effect due to PTT treatment. Moreover, the Ce_6_@PEICuS NPs are able to produce photoacoustic signals, thus making them promising for image-guided phototherapy, considering that they are not cytotoxic for the cells in the absence of irradiation [79].

**Figure 8:**
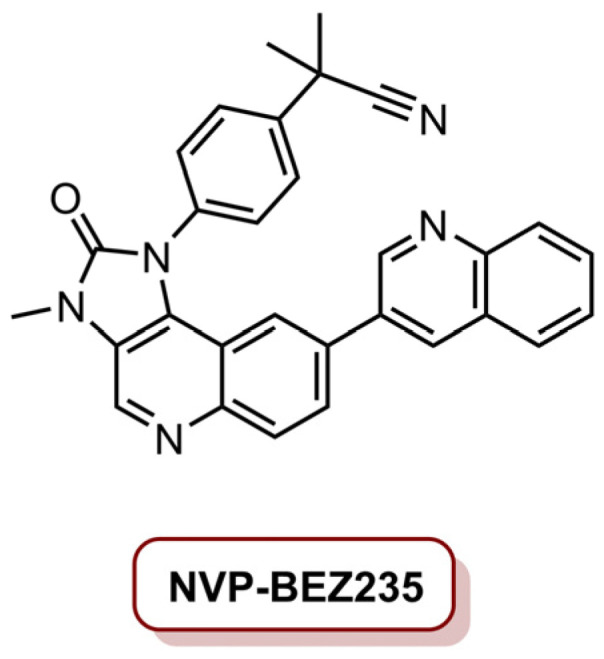
Structure of the dual PI3K-mammalian target of rapamycin (mTOR) inhibitor NVP-BEZ235.

In 2018, Eltahan et al. [23] developed a dual drug-loaded NVP/Ce_6_@NPs able to induce antitumour activity in MDA-MB-231 cancer cells. In this work, polymeric nanoparticles PEGylated poly(d,l-lactide-*co*-glycolide) (PLGA) were loaded with Ce_6_ and NVP-BEZ235, resulting in NVP/Ce_6_@NPs (Figure 8). NVP-BEZ235 has been studied in TNBC due to its ability to inhibit the PI3K/AKT/mTOR pathway and DNA damage repair in tumour cells [98]. The NVP/Ce_6_@NPs were prepared in concentrations of 75 and 25 µg mL^−1^, respectively, of NVP-BEZ235 and Ce_6_ [23]. The cells were incubated for 4 h and then irradiated with a laser at 660 nm during 10 min at an irradiance of 10 mW cm^−2^ (total light dose of 6.0 J cm^−2^). Following 48 h of dark incubation, NVP/Ce_6_@NPs showed higher toxicity than NPs loaded only with Ce_6_ (Ce_6_@NPs) or NVP-BEZ235 (NVP@NPs), and after only 24 h post-treatment, the effect of NVP/Ce_6_@NPs was similar to Ce_6_@NPs, which the authors interpreted as the toxicity of NVP/Ce_6_@NPs being mostly due to biochemical/PDT synergistic effect only after 48 h of incubation. Regarding the mechanism behind the photoactivation, the literature suggests that apoptosis is induced in cells treated with NVP/Ce_6_@NPs, due to DNA damage by the production of intracellular ROS, and the release of NVP-BEZ235, which allows the inhibition of DNA damage repair, resulting in an enhanced PDT efficiency. In vivo studies in a subcutaneous xenograft mouse model with MDA-MB-231 cells demonstrated that the tumour size was significantly reduced (89.3%) when treated with NVP/Ce_6_@NPs (4.0 mg kg^−1^ Ce_6_, 10 mg kg^−1^ NVP-BEZ235) and laser irradiation at an irradiance of 1.0 W cm^−2^ (total light dose of 1.8 kJ cm^−2^), supporting the biochemical/PDT synergistic effect [23].

**Figure 9:**
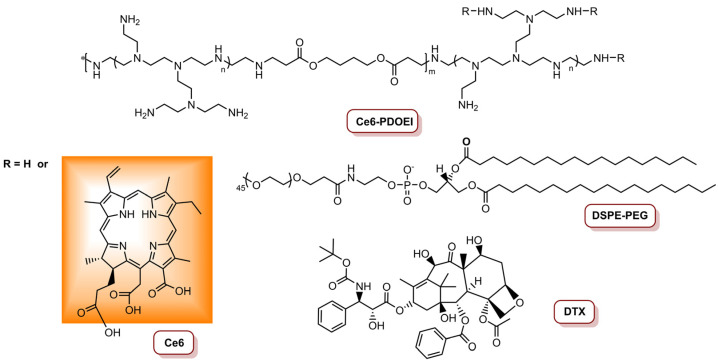
Representation of the structure of Ce_6_-PDOEI, DSPE-PEG and DTX involved in the preparation of CDTN.

Li and colleagues [80] reported the preparation of a Ce_6_-, docetaxel- (DTX) and anti-Twist siRNA-containing nanoparticle (CDTN) able to exert combined PDT/Chemotherapy/siRNA against 4T1 breast cancer cell line. This CDTN is constituted of a poly-β-aminoester derivative Ce_6_-grafted poly[(1,4-butanediol)-diacrylate-β-oligoethyleneimine_600_] (Ce_6_-PDOEI), 1,2-distearoyl-*sn*-glycero-3-phosphoethanolamine-*N*-[maleimide(polyethyleneglycol)_5000_] (DSPE-PEG), DTX (Figure 9) and an anti-Twist siRNA and was shown to be 50% more efficient in ^1^O_2_ production than DSPE-PEG free NPs. Additionally, significant fragmentation of siRNA after irradiation of the CDTNs was not observed, and the Twist-silencing activity had the highest activity at 0.11 W cm^−2^_._ The authors assessed the cytotoxicity and Twist-silencing efficiency of dual modality NPs in a mouse 4T1 cell line at different light conditions, i.e., mimicking superficial tumours 450 mW cm^−2^ (total light dose of 27 J cm^−2^) and deep tumours 110 mW cm^−2^ (total light dose of 6.6 J cm^−2^). The results revealed that the anti-cancer activity of CDTNs was spatially dependent, i.e., PDT was responsible for killing cancer cells in the superficial part of the tumour and the inhibition of the metastasis of residual cells was achieved via reduction in Twist expression with siRNAs. On the other hand, cancer cells were killed through PDT-potentiated chemotherapy, and metastasis of mesenchymal-like cancer cells via PDT-potentiated Twist downregulation in deep areas of the tumour tissue [80].

**Figure 10:**
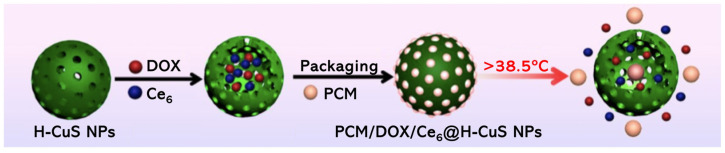
Representation of the thermo-responsive nanostructure, based on hollow mesoporous copper sulphide NPs (PCM/DOX/Ce6@H-CuS NPs), for trimodal therapy (reproduced from Reference [81] with permission from the American Chemical Society).

Li et al. [81] reported a thermo-responsive nanostructure, based on hollow mesoporous copper sulfide NPs (H-CuS NPs), for trimodal therapy. DOX and Ce_6_ were encapsulated inside the H-CuS NPs via drug-loaded phase change material (PCM), resulting in PCM/DOX/Ce_6_@H-CuS NPs (Figure 10). The PCM used was the biocompatible and FDA-approved food and cosmetic additive 1-tetradecanol, that is known to control the release of organic dyes from hollow polymer particles at 39 °C [99,100]. The different entities implicated in the preparation of these NPs allow the combination of different therapies, i.e., chemotherapy, PDT and PTT [81]. PCM/DOX/Ce_6_@H-CuS NPs were able to internalize in 4T1 cells, as was observed by fluorescence signals of Ce_6_ and DOX in the cytoplasm. Biological evaluation of such NPs at different concentrations (5.0, 10, 20, 50, 100 and 200 µg mL^−1^) revealed that when PDT (660 nm, 0.5 W cm^−2^, 150 J cm^−2^) was performed for 5 min, no cytotoxicity was observed; however, when PTT (808 nm, 2.0 W cm^−2^, 600 J cm^−2^) was performed for the same 5 min, a significant decrease in cell viability was noted, and this observation was related with the occurrence of hyperthermia and release of DOX from the melted PCM. Moreover, when the PDT and PTT were combined, it was observed that the decrease in cell viability was different if the order of the applied therapies changed, i.e., the better efficiency was found when PTT was performed prior to PDT, once the Ce_6_ and DOX are released and stay able to the photodynamic and chemotherapeutic effects, respectively. In vivo studies on BALB/c nude mouse models carrying 4T1 tumours revealed a 98.4% tumour inhibition rate after 14 days post-injection with the PCM/DOX/Ce_6_@H-CuS NPs (DOX dosage 2.0 mg kg^−1^ and Ce_6_ dosage 5.0 mg kg^−1^) and laser at 808 nm (2.0 W cm^−2^, 600 J cm^−2^), plus laser at 660 nm (0.5 W cm^−2^, 150 J cm^−2^). This trimodal therapy induced tumour cell apoptosis, leading to tumour inhibition both in vitro and in vivo. Additionally, this thermo-responsive nanostructure revealed good biocompatibility and minimal systemic toxicity [81].

In 2019, Zhou et al. [82] developed a prolonged oxygen-generating phototherapy hydrogel (POP-Gel) through the combination of the Ce_6_-loaded hydrogel with CaO_2_ and catalase (CAT). In this system, CaO_2_ slowly reacts with water to produce H_2_O_2_, which is decomposed by CAT in the hydrogel to produce dioxygen for up to 120 h. POP-Gel was able to produce ^1^O_2_, exhibiting a twofold enhancement in fluorescence, when compared with the hydrogel without CaO_2_ and CAT (P-Gel). After 24 h of incubation, both gels entered into 4T1 tumour spheroids and dark toxicity was not observed. However, when 4T1 cells were exposed to light at an irradiance of 5.0 mW cm^−2^ at 660 nm during 30 min (total light dose of 9.0 J cm^−2^), POP-Gel demonstrated high toxicity, with an IC_50_ of 0.359 µg mL^−1^, when compared with P-Gel with an IC_50_ of 1.444 µg mL^−1^. POP-Gel also presented the ability to produce higher intracellular ROS and to induce higher levels of apoptosis and/or necrosis at a concentration of 0.5 µg mL^−1^ (in terms of Ce_6_ content). Under hypoxia, the POP-Gel drastically reduced the levels of hypoxia-inducible factor (HIF)-1α in 4T1 cells. In vivo studies revealed that Ce_6_ mainly accumulated in the tumour, and both gels retained the Ce_6_ loads and accumulated around the tumour for up to 7 days; additionally, POP-Gel was able to be in the injection site for more than 5 days and increased the oxygenation levels from 22% to 66%. The results of therapeutic effect in 4T1 tumour-bearing mice showed that when POP-Gel was injected around the tumour, the Ce_6_ was able to penetrate the tumour and reduce the expression of HIF-1α to low levels after PDT treatment. The observation of DNA fragmentation and the high percentage of TUNEL-positive cells also highlights that POP-Gel-mediated PDT induces apoptosis and/or necrosis. Regarding the tumour progression after 16 days of initial treatment, POP-Gel, at a concentration of 1.0 mg mL^−1^, when irradiated with 100 mW cm^−2^ (on days 1, 3 and 5), caused the biggest reduction in tumour volumes and their growth slowed, when compared with P-Gel. Additionally, no severe systemic toxicity was observed. Moreover, treatment with POP-Gel causes a decrease in HIF-1α and VEGF levels and inhibited the metastasis of the primary tumour to inguinal lymph nodes; additionally, after PDT treatment in orthotopic 4T1 tumour-bearing mice, the primary tumours growth was supressed, and the metastasis to the lung was reduced to only 3%. In conclusion, the results showed that, after PDT treatment, POP-Gel downregulates the expression of HIF-1α and VEGF once there is sufficient production of dioxygen, consequently causing the inhibition of tumour growth and metastasis [82].

**Figure 11:**
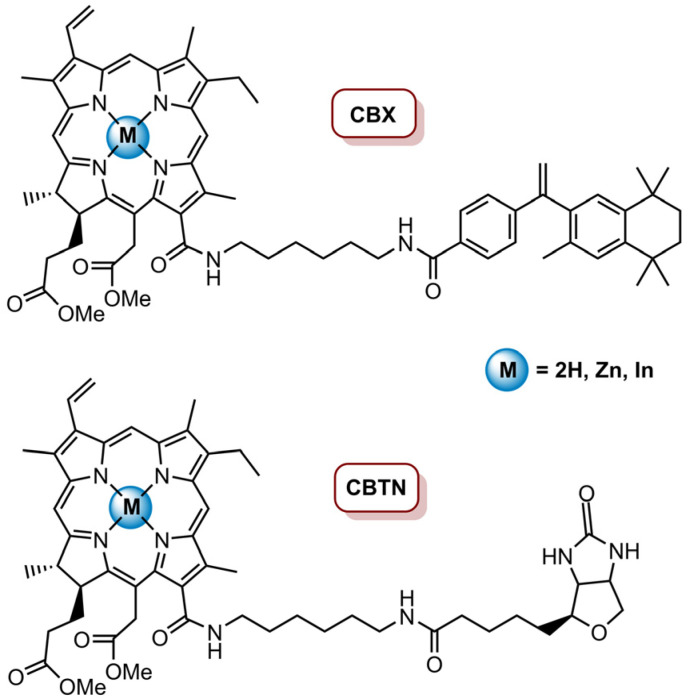
Transporter-targeted drug delivery systems, CBX and CBTN, and their corresponding Zn(II) and In(II) complexes.

Mee and Isaac-Lam [83] prepared transporter-targeted drug delivery systems (CBTN and CBX) through the conjugation of methyl pheophorbide *a* (MePheo) with biotin (BTN) and bexarotene (BX) and their corresponding zinc(II) and indium(II) complexes (Figure 11). These drug delivery systems aimed to target the vitamin receptors (biotin receptors) and the nuclear receptors (retinoid X receptors), which are overexpressed in cancer cells CBTN and CBX, respectively, and were tested against TNBC cell line BT-549. In vitro studies revealed that CBTN, Zn-CBTN, In-CBTN and In-CBX had some dark toxicity at higher concentrations (>5.0 µM). However, no dark toxicity for all systems at concentrations lower than or equal to 0.5 µM was observed; thus, the phototoxic effect in BT-549 was tested at a concentration of 0.5 µM and cells were irradiated with a LumaCare LC-122 650 nm for 1 min (0.96 J cm^−2^), 2 min (1.92 J cm^−2^) and 5 min (4.8 J cm^−2^) at an irradiance of 0.016 W cm^−2^. It was revealed that CBTN (after a light dose of 0.96 J cm^−2^) causes a 77% decrease in cancer cells proliferation, CBX only causes a 16% decrease and with MePheo, no effect in cell proliferation was observed. Regarding Zn and In complexes of both CBTN and BTX, no phototoxic effect was observed. The derivatives were also tested at different concentrations (1.0, 5.0, 10, 25 and 50 µM) with a light dose of 0.96 J cm^−2^ and a more evident dose response was observed for all derivatives, except for Zn-CBX. Additionally, CBTN was shown to be the most efficient in cellular growth inhibition, followed by CBX and then MePheo. ZnCBTN and InCBTN revealed to be more effective than In-CBX and Zn-CBX. The systems phototoxicity at nanomolar concentrations was also assessed, and the results demonstrated that CBTN at 100 nM causes a 60% cell inhibition after irradiation with a total light dose of 4.8 J cm^−2^. The better photodynamic efficiency observed for CBTN was associated with the fact that the biotin unit was more hydrophilic than bexarotene, which makes CBTN much more polar. Additionally, the higher amphiphilicity of CBTN would enhance the intracellular uptake, improving the photodynamic efficiency. Regarding the PDT mechanisms, it was observed that, at a concentration of 5.0 µM and a total light dose of 1.96 J cm^−2^, the cells treated with CBTN, InCBTN, MePheo and InCBX presented morphological apoptosis characteristics, such as nuclear fragments and chromatin condensation; additionally, there was no indication that necrotic or autophagic pathways were responsible for cell death mechanisms, thus apoptosis seems to be the mechanism behind the photoactivation of these delivery systems by PDT [83].

**Figure 12:**
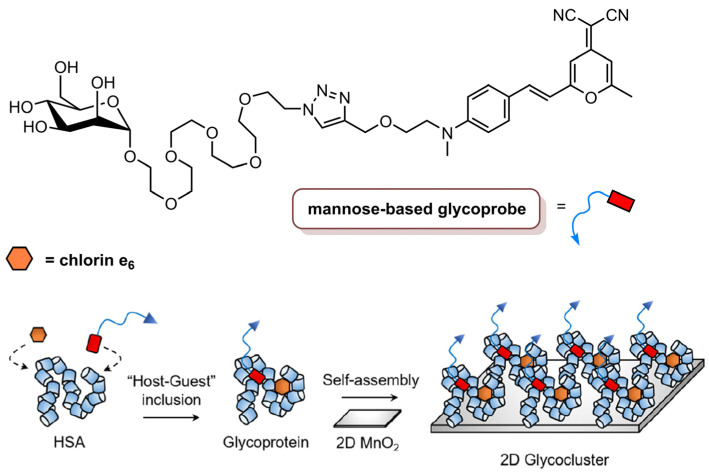
Schematic representation of the inclusion of the glycoprobe and Ce_6_ to HSA, resulting in the glycoprotein that was self-assembled with 2D MnO_2_ for the formation of 2D glycocluster (reproduced from Reference [84] with permission from the American Chemical Society).

He and co-workers [84] reported the synthesis of a two-dimensional glycocluster, based on 2D MnO_2_, for the targeted delivery of theranostic agents (Figure 12). The synthesis involved the formation of the mannose-based glycoprobe through click conjugation between a PEG-linked azido-mannoside and an alkynyldicyanomethylene-4*H*-pyran dye. Then, human serum albumin (HSA), which has hydrophobic pockets that are able to attach to small molecules, was used to encapsulate the glycoprobe and the Ce_6_ for further self-assembling with 2D MnO_2_, which can be degraded by the biothiols inside cells. The 2D glycocluster had a similar formation of ROS at 660 nm as Ce_6_ alone. The MDA-MB-231 cell line was chosen for biological studies, since, besides overexpressing mannose receptors, it contains high enough levels of endogenous glutathione for degradation of 2D MnO_2_, and a control cell line, HeLa, that also contains glutathione but does not express mannose receptors. Fluorescence microscopy studies showed that 2D glycocluster enhanced the fluorescence of Ce_6_ in MDA-MB-231 cells but not in HeLa cells; thus, the mannose group in the 2D glycocluster seems to be recognized by mannose receptors in the cancer cells, which allows the active endocytosis of the 2D glycocluster. The photodynamic efficiency of Ce_6_ (1.0 µM), Ce_6_@HSA (1.0 µM Ce_6_/40 µM HSA), Ce_6_@HSA@2D MnO_2_ (1.0 µM Ce_6_/40 µM HSA/30 µg mL^−1^ 2D MnO_2_) and 2D glycocluster (10 µM glycoprobe/1.0 µM Ce_6_/40 µM HSA/30 µg mL^−1^ 2D MnO_2_) was evaluated at an irradiance of 1.0 W cm^−2^ with red light at 660 nm for 15 min (total light dose of 900 J cm^−2^). The viability of MDA-MB-231 was decreased by 2D glycocluster but remained unaltered when the glycoprobe was absent. Additionally, the viability of HeLa cells was not affected by any of the tested materials. To corroborate the targetability of the 2D glycocluster, another TNBC cell line (MDA-MB-468) was used, and a similar result was observed—the 2D glycocluster targeted cancer cells and was more effective in reducing cancer cells viability. Nevertheless, MDA-MB-231 cells, which have higher expression levels of mannose receptors, were the most sensitive to the treatment. In vivo fluorescence imaging in a xenograft mouse model showed that the accumulation of Ce_6_, derived by the 2D glycocluster in the tumour, was much stronger that of Ce_6_ alone. These results revealed the promise of this 2D glycocluster for targeted imaging and therapy in TNBC [84].

**Figure 13:**
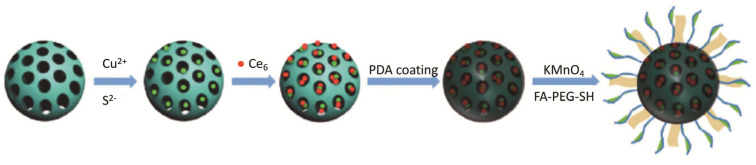
Representation of the nano-theranostic formulation for MRI and PTT/PDT dual therapy (reproduced from Reference [85] with permission from Springer Nature).

Recently, Zhang et al. [85] reported the development of a nano-theranostic formulation for MRI and PTT/PDT dual therapy. In this work, CuS was immobilized in the thiol-functionalized mesoporous silica nanoparticles (MSN NPs). Then, Ce_6_ was loaded into the CuS/MSN NPs and these were encapsulated with polydopamine (PDA). Finally, PDA was functionalized with MnO_2_ nano-sheet and conjugated with tumour-targeting ligand folic acid–PEG–thiol (FA–PEG–SH), resulting in the Ce_6_-CuS/MSN@PDA@MnO_2_-FA NPs (Figure 13). This formulation revealed low dark cytotoxicity until the maximum concentration tested (200 µg mL^−1^) and high cellular uptake by 4T1 cells. Photocytotoxic studies showed that treatment with Ce_6_-CuS/MSN@PDA@MnO_2_-FA NPs ([Ce_6_] = 16 µg mL^−1^ and [CuS] = 60 µg mL^−1^) and combined PTT (808 nm, 2.0 W cm^−2^, 1.2 kJ cm^−2^, 10 min) and PDT (660 nm, 50 mW cm^−2^, 30 J cm^-2^, 10 min) reduced the cell viability to 2%. Strong MRI signals, 12h post-injection, confirmed Ce_6_-CuS/MSN@PDA@MnO2-FA NPs accumulation inside 4T1 tumour cells. Moreover, in vivo experiments in a 4T1 tumour-bearing mice treated with Ce_6_-CuS/MSN@PDA@MnO2-FA NPs and combined therapy revealed that, after 14 days of treatment, the tumours almost disappeared due to nuclear shrinkage and cytoplasm leakage in apoptotic cells [85].

**Figure 14:**
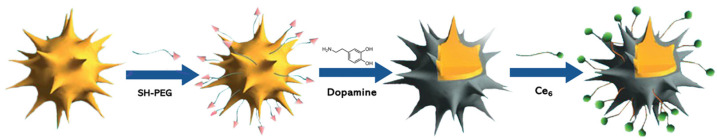
Representation of Ce_6_-conjugated and polydopamine-coated gold nanostars (Ce_6_-PDA@AuNSs) (reproduced from Reference [86] with permission from the Royal Society of Chemistry).

In another study, Zeng and co-workers [86] reported a Ce_6_-conjugated and polydopamine (PDA)-coated gold nanostars (AuNSs) for PTT/PDT and photoacoustic imaging. The preparation of this material involved the deposit of PDA on the surface of AuNSs by dopamine self-polymerization, resulting in PDA@AuNSs. Then, Ce_6_ was conjugated to PDA@AuNSs through covalent conjugation, leading to the formation of Ce_6_-PDA@AuNSs (Figure 14). In vitro studies in 4T1 cells, at a concentration of 50 µg mL^−1^, demonstrate that laser irradiation at 808 nm (5 min, 1.0 W cm^−2^, 300 J cm^−2^) reduced the cell viability to only 14.7%; however, when PDT treatment was performed (635 nm laser, 5 min, 50 mW cm^−2^, 15 J cm^−2^) the reduction was slight lower, with cell viability reduced to 28.2%. In turn, combined PTT/PDT, at the same concentration, reduced the cell viability to practically zero. A 4T1 tumour-bearing nude mice model was used for in vivo studies, and it was observed that when the animal was treated with Ce_6_-PDA@AuNSs (200 µg mL^−1^) and single laser irradiation (808 nm or 635 nm), at 15 days post-injection the tumour growth was partially inhibited, but when PTT and PDT were combined, the tumour disappeared. Moreover, combined PTT/PDT treatment was effective in the inhibition of lung metastasis, contrarily to PDT or PTT alone, where some lung metastasis was observed. Moreover, photoacoustic signals of tumours were observed after injection with Ce_6_-PDA@AuNSs, which emphasises its potential simultaneous application as a phototheranostic agent [86].

**Figure 15:**
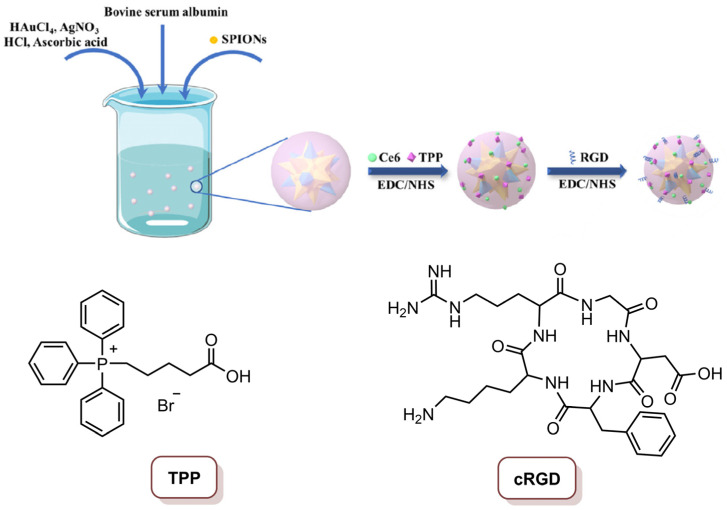
Schematic representation of the multifunctional magnetic gold nano-heterostructure loading Ce_6_ and functionalized with cRGD peptide and TPP molecule (Ce_6_@MGN@RT) (reproduced from Reference [22] with permission from Elsevier).

Xue and co-workers [22] reported a multifunctional magnetic gold nano-heterostructure (MGN)-loading Ce_6_ (Ce_6_@MGN), functionalized with the cRGD cell membrane-targeting peptide and the mitochondria-targeting 4-carboxybutyl)triphenylphosphonium (TPP) molecule for synergistic photothermal and photodynamic therapy (Figure 15). This cell membrane/mitochondria, dual-targeted MGN, was prepared through a facile seed-mediated synthetic method and superparamagnetic iron oxide nanoparticles (SPIONS) were used as the seed. Subsequently, Ce_6_ was loaded by covalent coupling on the MGN, and the loading ratio of Ce_6_ on the MGN was 78.6%. The last step was the functionalization of the MGN with cRGD (to target α_v_β_3_ integrin at the cancer cell membrane) and TPP (to target the mitochondria), to obtain the multifunctional Ce_6_@MGN@RT. The characterization of this Ce_6_@MGN@RT revealed its excellent stability in water and photothermal conversion capacity. Additionally, Ce_6_@MGN@RT were able to produce ^1^O_2_ after laser irradiation at 660 nm, which makes this nanosystem a good candidate for the combined PTT and PDT application. In vitro studies of confocal microscopy revealed that dual-targeting methodology improved the targeting skill of the multifunctional Ce_6_@MGN@RT towards MBA-MD-231 cells. This observation was suggested to be due to the fact that the recognition of the nanosystem by the cell membrane is aided by the cRGD-coupled moiety; additionally, on the other hand, the TPP binding allowed the nanosystem to evade lysosomes to mitochondria, which was confirmed by the lysosome’s destruction. The therapeutic effect of Ce_6_@MGN@RT showed insignificant cytotoxicity at concentrations lower than 40 µg mL^−1^ (in terms of Au content), but when the combined PTT (808 nm laser; 0.8 W cm^−2^; 3 min; 144 J cm^−2^) and PDT (660 nm laser, 0.03 W cm^−2^; 3 min; 5.4 J cm^−2^) were performed, it was possible to observe an 81% reduction in cell viability at a concentration of 20 µg mL^−1^. In turn, when PTT and PDT were performed separated, a lower efficiency in the anti-cancer effect toward cancer cells was observed. The generation of ROS, and the high effect of hyperthermia after irradiation at 660 nm and 808 nm, respectively, could be the mechanisms for the anti-cancer activity, since this combined therapy can trigger serious dysfunction of intracellular mitochondria and then the apoptosis of cells. The efficiency of this combined therapy in vivo revealed that Ce_6_@MGN@RT (injected and subjected to the laser treatment every 2 days) could target the tumours; additionally, more interestingly, it exhibited significant tumour growth inhibition in MBA-MD-231 cancer xenografted mouse model after 21 days of treatment by intra-tumoral injection and intravenous injection. Apparently, the intravenous injection has slightly lower efficiency in inhibiting tumour growth; however, the ability of targeting tumours still causes this procedure to present with outstanding tumour inhibition. In fact, this biocompatible material showed synergistic effect after combined PTT and PDT, by triggering apoptosis of tumour cells and consequently provoking tumour growth suppression [22].

The studies involving chlorin photoactive compounds/formulations, used in different therapeutic modalities, are summarized in Table 1 (PDT, PDT/PTT, PDT/Chemotherapy and its combinations). The table highlights the light conditions, the cell lines and the main findings of each study.

## 5. Final Remarks

TNBC is a highly aggressive disease, with no targeted therapy options and worse survival rate. Therefore, ongoing efforts aim to improve treatment options. In this sense, important aspects need to be considered for the development of safe and effective therapies and strategies. Throughout this review, it was noted that the knowledge of the presence of several receptors in cancer cells allows the development of specific targeted therapies against TNBC. In this context, in recent years, the scientific community has been reporting the preparation of chlorin-based photoactive molecules, coupled with specific targets and/or drug delivery systems and with other anti-cancer drugs, for effective therapies against TNBC. Among the several strategies using chlorin-based formulations discussed in this review, it was noticed that the most common is the combination of PDT with chemotherapy or PTT with PDT. These dual therapies demonstrated good efficacies in the reduction in TNBC cells and in the inhibition of tumour growth. At least one study that combines these three therapies (PTT/PDT/chemotherapy) was also found, which revealed the combination to be a promising strategy against TNBC both in vitro and in vivo—this can be further explored. Additionally, therapies that aim to target different receptors present in cancer cells, such as EGFR, biotin receptors and mannose receptors, were explored and these revealed encouraging results. The interesting results found in the studies on photodynamic targeted therapy seem to be related to the production of ROS by the PS molecule, the targeting ability of the molecules and the synergistic effect of combined therapies, such as PTT/PDT and PDT/chemotherapy. In the case of PTT/PDT, the thermal effect of PTT was shown to be responsible for the thermal ablation of tumours by hyperthermia; additionally, it was shown to help the release of photosensitizers from the nanoparticles, so they can be available for photodynamic process. On the other hand, in combined PDT/chemotherapy, the photodynamic effect was also improved thanks to the chemotherapeutic agents present in the formulations. The presence of morphological characteristics of apoptosis in some of the reported studies indicates that this is the main mechanism of cell death that occurs by photodynamic targeted therapy. However, there are still some issues related to the clearance of these formulations from the organism and concerning the degradation products. Moreover, most of the studies discussed in this review used Ce_6_ formulations, and although their efficient photodynamic action is well known, since it has been extensively explored in several PDT studies, there are other chlorin-based molecules that could be exploited.

Although, in this review, only studies with chlorin-based formulations were discussed, there are several studies in the literature with other PS molecules (e.g., porphyrins, phthalocyanines and dyes, among others), coupled, or not coupled, to other targets and delivery systems. These have been revealed to be quite promising in therapies targeting TNBC, and as diagnostic tools. Such studies indicate the growing concern in finding an effective targeted therapy that improves the prognosis in this type of BC. The fact that chlorin-based molecules are available from natural sources is relevant, since they may allow for a better biocompatibility and rapid clearance from the body.

## Figures and Tables

**Table 1:** Summarized studies with the different chlorin photoactive compounds/formulations.

Photoactive Compund/ Formulation	Chlorin Molecule	Cell Lines	Therapeutic Modality	Light Conditions	Main Findings (IC_50_ or % Cell/Tumour Inhibition)	Ref.
RedoxT	Ce_4_	MDA-MB-231 and MDA-MB-468	PDT	670 nm CW laser, 50 W cm^−2^ (20 J cm^−2^)	IC_50_ of 0.66 µM in MDA-MB-468 cells IC_50_ of 1.80 µM in MDA-MB-231 cells	[75]
NVP/Ce_6_@NPs	Ce_6_	MDA-MB-231		660 nm laser, 1000 mW cm^−2^ (1.8 kJ cm^−2^)	89.3% tumour size reduction (4.0 mg kg^−1^ Ce_6_, 10 mg kg^−1^ NVP-BEZ235)	[23]
2D glycocluster				660 nm laser, 1000 W cm^−2^ (900 J cm^−2^)	Cell viability decreased in the presence of glycoprobe (10 µM glycoprobe/1.0 µM Ce_6_/40 µM HSA/30 µg mL^−1^ 2D MnO_2_)	[84]
POP-Gel		4T1		660 nm laser, 5.0 mW cm^−2^ (9.0 J cm^−2^)	IC_50_ of 0.359 µg mL^−1^	[82]
CBTN and CBTX	MePheo	BT-549		650 nm LumaCare LC-122, 16 mW cm^−2^ (0.96 J cm^−2^, 1.92 J cm^−2^, 4.8 J cm^−2^)	60% cell inhibition for CBTN at 100 nM (4.8 J cm^−2^); 77% decrease in cancer cells proliferation for CBTN; 16 % decrease for CBX at 0.5 µM (0.96 J cm^−2^)	[83]
EAT@NPs	Ce_6_	MDA-MB-231	PDT/Chemo	670 nm CW laser, 68 mW cm^−2^ (10 J cm^−2^)	98% cell inhibition (20 μM CPT equivalent)	[74]
ELTSL-HOC/DOX	PPa	MDA-MB-231, MDA-MB-468 and 4T1		670 nm laser, 400 mW cm^−2^ (240 J cm^−2^)	Supressed the growth of the tumour in an orthotopic 4T1 tumour model	[77]
RADV		4T1		670 nm laser, 100 mW cm^−2^ (12 J cm^−2^) in vitro; 200 mW cm^−2^ (60 J cm^−2^) in vivo	In vitro: 87.5% cell inhibition (DOX concentration of 1.0 µM) In vivo: 85% tumour inhibition (DOX dosage of 5.0 mg kg^−1^)	[78]
iNP@AC	Acid derivative of Ce_6_ (AC)			655 nm laser, 250 mW cm^−2^ (7.5 J cm^−2^), 630 mW cm^−2^ (18.9 J cm^−2^)	81% cell inhibition (concentration of 300 nM platinum and 8.0 nM AC)	[76]
Ce_6_@PEICuS NPs	Ce_6_	MDA-MB-231	PDT/PTT	670 nm laser, 100 mW cm^−2^ (60 J cm^−2^); 808 nm laser, 2.0 W cm^−2^ (1.2 kJ cm^−2^)	PDT: 55% cell inhibition; PTT: 41% cell inhibition; PDT/PDT: 84% cell inhibition (200 µg mL^−1^)	[79]
Ce_6_@MGN@RT				660 nm laser, 30 mW cm^−2^ (5.4 J cm^−2^); 808 nm laser, 0.8 W cm^−2^ (144 J cm^−2^)	81% cell inhibition after PDT/PTT (20 µg mL^−1^)	[22]
Ce_6_-CuS/MSN@PDA@MnO_2_-FA NPs		4T1		660 nm laser, 50 mW cm^−2^ (30 J cm^−2^); 808 nm laser, 2.0 W cm^−2^ (1.2 kJ cm^−2^)	2% of cell viability (16 µg mL^−1^ of Ce_6_ and 60 µg mL^−1^ of CuS)	[85]
Ce_6_-PDA@AuNSs				635 nm laser, 50 mW cm^−2^ (15 J cm^−2^); 808 nm laser 1.0 W cm^−2^ (300 J cm^−2^)	PDT: reduction to 28.2%; PTT: reduction to 14.7%; PDT/PTT: reduction to approximately zero in cell viability (50 µg mL^−1^); the tumour disappeared after PDT/PTT treatment (200 µg mL^−1^)	[86]
CDTN	Ce_6_	4T1	PDT/Chemo/siRNA	671 nm laser, 450 mW cm^−2^ (27 J cm^−2^); 110 mW cm^−2^ (6.6 J cm^−2^)	Cancer cells were killed in superficial tumours via PDT, and in deep tumours via PDT-potentiated chemotherapy and Twist downregulation	[80]
PCM/DOX/Ce_6_@H-CuS NPs	Ce_6_	4T1	PDT/PTT/Chemo	660 nm laser, 500 mW cm^−2^ (150 J cm^−2^); 808 nm laser, 2.0 W cm^−2^ (600 J cm^−2^)	98.4% tumour inhibition (DOX dosage 2.0 mg kg^−1^ and Ce6 dosage 5.0 mg kg^−1^)	[81]

## Data Availability

This study did not report any data.
